# The *Global strategy for women’s, children’s and adolescents’ health (2016–2030)*: a roadmap based on evidence and country experience

**DOI:** 10.2471/BLT.16.170431

**Published:** 2016-05-02

**Authors:** Shyama Kuruvilla, Flavia Bustreo, Taona Kuo, CK Mishra, Katie Taylor, Helga Fogstad, Geeta Rao Gupta, Kate Gilmore, Marleen Temmerman, Joe Thomas, Kumanan Rasanathan, Ted Chaiban, Anshu Mohan, Anna Gruending, Julian Schweitzer, Hannah Sarah Dini, John Borrazzo, Hareya Fassil, Lars Gronseth, Rajat Khosla, Richard Cheeseman, Robin Gorna, Lori McDougall, Kadidiatou Toure, Kate Rogers, Kate Dodson, Anita Sharma, Marta Seoane, Anthony Costello

**Affiliations:** a World Health Organization, avenue Appia 20, 1211 Geneva 27, Switzerland.; b Executive Office of the United Nations Secretary-General, Every Woman Every Child Health Team, New York, United States of America (USA).; c Ministry of Health and Family Welfare, Government of India, New Delhi, India.; d United States Agency for International Development, Government of the United States of America, Washington, USA.; e Norwegian Agency for Development Cooperation, Government of Norway, Oslo, Norway.; f United Nations Children’s Fund, New York, USA.; g United Nations Office of the High Commissioner for Human Rights, Geneva, Switzerland; h Aga Khan Development Network, Nairobi, Kenya.; i Partners in Population and Development, Dhaka, Bangladesh.; j Results for Development, Washington, USA.; k Robert Taylor Communications, London, England.; l Partnership for Maternal, Newborn & Child Health, Geneva, Switzerland.; m United Nations Foundation, New York, USA.

The *Global strategy for women’s, children’s and adolescents’ health (2016–2030) *provides a roadmap for ending preventable deaths of women, children and adolescents by 2030 and helping them achieve their potential for and rights to health and well-being in all settings.^1^ The global strategy has three objectives: survive (end preventable deaths); thrive (ensure health and well-being); and transform (expand enabling environments). These objectives are aligned with 17 targets within nine of the sustainable development goals (SDGs),^2^ including SDG 3 on health and other SDGs related to the political, social, economic and environmental determinants of health and sustainable development. 

Like the SDGs, the global strategy is universal in scope and multisectoral in action, aiming for transformative change across numerous challenging areas for health and sustainable development (Box 1).^1^ The strategy was developed through evidence reviews and syntheses and a global stakeholder consultation,^3^^,^^4^ and draws on new thinking about priorities and approaches for health and sustainable development.^4^ Particular attention was given to experience gained and lessons learnt by countries during implementation of the previous *Global strategy for women’s and children’s health (2010–2015)*^5^ and achieving the millennium development goals (MDGs).^6^^,^^7^ A five-year operational framework with up-to-date technical resources has also been developed to support country-led implementation of the global strategy. This framework will be regularly updated until 2030.^1^^,^^3^

Box 1 **The *Global strategy for women’s, children’s and adolescents’ health (2016–2030)***

**Objectives of the global strategy:**

- Survive: end preventable mortality;
- Thrive: promote health and well-being; and
- Transform: expand enabling environments.

**Five drivers of change to achieve the objectives based on the global strategy action areas:**

- People: individual potential and community engagement;
- Political effectiveness: country leadership, financing, accountability;
- Programmes: health system, multisector, humanitarian, research and innovation;
- Partnerships: Every Woman Every Child Partnerships, including the Global Financing Facility, the United Nations and multilateral H6 partnership, Unified Accountability Framework and Independent Accountability Panel, Innovation Marketplace and other national, regional and global partnerships; and
- Principles: country-led, universal, sustainable, human-rights based, equity-driven, gender-responsive, evidence-informed, partnership-driven, people-centred, community-owned, accountable, aligned with development effectiveness and humanitarian norms.

Evidence shows that progress is required across a set of overlapping and mutually reinforcing areas to improve the health, dignity and well-being of women, children and adolescents.^4^^,^^7^^,^^8^ Key areas for action were set out in the first global strategy (2010–2015), including health financing; the health system and workforce; access to essential interventions and life-saving commodities; national leadership; and accountability.^5^ Based on emergent evidence, sociopolitical and environmental changes and the SDGs, the current global strategy (2016–2030) includes new strategic areas, for example adolescent health; humanitarian and fragile settings; an integrated life-course approach to health recognizing the links across different stages; multisector approaches; and guiding principles such as universality, human rights, equity and development effectiveness.^1^

Evidence indicates that countries can accelerate progress in health and sustainable development through integrated action within the health sector and across social, economic and environmental sectors.^7^^,^^9^ For example, through investments across sectors, the Chinese government lifted 439 million people out of poverty between 1990 and 2015, reduced child and maternal mortality by over 80% and 72%, respectively, and raised secondary school enrolment to over 99%, with equal numbers of boys and girls enrolled. Rural access to clean water and sanitation also improved to over 85% and 74%, respectively.^10^^–^^12^ In Ethiopia, a similar approach reduced poverty from 48% in 1990 to 23% in 2015, and the country experienced improvements in education, roads, water, sanitation and hygiene. Over the same period, child and maternal mortality declined by 71% and 72%, respectively.^7^^,^^11^^,^^12^

The actions and approaches required to achieve the objectives of the global strategy (2016–2030)^1^ converge around five main drivers of change: people; political effectiveness; programmes; partnerships; and principles. The following sections highlight how some countries have already begun achieving these transformative changes (Box 1).

The global strategy (2016–2030) emphasizes the importance of measures to help all women, children and adolescents to realize their rights and full potential for health and well-being. These measures include policies and programmes for early childhood development and adolescent health. Removing barriers to enjoyment of rights– such as those to gender-equality and women’s socioeconomic and political participation are also important measures.^1^

Evidence shows that early childhood development programmes have significant long-term health and socioeconomic advantages. Parenting resources for early childhood development, school-community outreach and health services have measureable physical, intellectual and socioeconomic benefits for children, their families and communities. Such actions can reduce health, special schooling and criminal justice expenditures.^13^

Healthy, educated adolescents can better realize their potential, contribute to the demographic dividend and economic growth, as seen in east Asia in the 1980s and 1990s.^14^ Evidence shows that with investment and political commitment for adolescent health and development, rapid progress can be made.^15^ Now, countries such as Argentina, Colombia, Estonia, Ethiopia, India, the Republic of Moldova, Senegal and Uganda are investing in large-scale adolescent health and development programmes to gain similar dividends. Investments could help countries in sub-Saharan Africa realize annual dividends of at least 500 billion United States dollars (US$), equal to about one third of the region’s current gross domestic product, for as many as 30 years.^14^

Women’s social, political and economic participation is associated with better health outcomes for women and children.^7^ In Rwanda, where 64% of parliamentarians are women and where the parliament has committed to and invested in health and development, maternal and child mortality declined by 78% and 72%, respectively, between 1990 and 2015.^7^^,^^12^ At community level, women’s groups in Bangladesh, India, Malawi and Nepal contributed to better access to quality health services and improved maternal and newborn health.^16^

Leadership at all levels of society is a proven prerequisite for progress.^1^^,^^4^^,^^7^ In Kyrgyzstan, committed political leadership, clear policy, management capacity and low staff turnover in the health ministry contributed to sustained financing, improved health services and a reduction of child mortality by almost two thirds since 1990.^11^^,^^17^ Political effectiveness can also drive cross-sector action to address diverse determinants of health. Collaboration across sectors during the MDG era helped some countries to accelerate progress to reduce mortality, malnutrition and gender inequality, to strengthen health and education systems and to improve water quality, sanitation and infrastructure.^18^

Robust data and analysis are essential to enable accountability through a cycle of monitoring, independent review and action to ensure that programmes and policies are achieving their desired objectives. For example, in Mozambique a coalition of partners invested in the country’s civil registration and vital statistics system, increasing registered deaths by 18% from 2012 to 2014 and enabling routine reporting of causes of death by sex and age for the first time since 1975 (Commission on Information and Accountability, Mozambique, unpublished data, December 14, 2015).

To strengthen accountability, at least 50 countries with a high burden of maternal and child mortality had regular national health sector review processes that met basic accountability criteria in 2015, and another 36 countries had adopted the good governance for medicines approach to battle corruption.^19^^,^^20^

Quality programmes in health and other sectors, and for research and innovation, can catalyse change, even in humanitarian and fragile settings. While resilient health systems and universal coverage of quality care are gold standards for women’s, children’s and adolescents’ health, catastrophic events can swiftly undo hard-won health gains, particularly where existing health systems are weak. For example, during the 2013–2016 Ebola disease outbreak in Liberia, skilled birth attendance fell from 52% to 38%, vaccination rates dropped and 64% of health facilities were not operational.^21^

Experience shows that quality care is possible even under extreme circumstances. In Jordan, humanitarian and development partners have collaborated to give all residents of Za’atari refugee camp access to maternal and child health centres, while additional health centres serve Syrian refugees who are not living in camps.^22^ The global strategy (2016–2030) highlights the importance of expanding such collaborative practices and improving emergency preparedness at all levels of the health system.

While the health sector remains central for people’s health, there is evidence that in low- and middle-income countries about 50% of gains in women’s and children’s health since 1990 have resulted from progress in non-health sectors.^4^^,^^23^^,^^24^ Investments in nutrition, water and sanitation were essential in eradicating polio in India, which was certified as polio-free in 2014. Previous efforts, focused on vaccination alone, were insufficient because malnourishment and diarrhoea from unsafe water and inadequate sanitation limited vaccine effectiveness.^25^ Education is also critical to improving health and well-being. In Malawi, conditional cash transfers to encourage school attendance by girls were associated with reductions in teenage pregnancies, early marriage and human immunodeficiency virus infections.

Evidence shows that knowledge and innovation are at least as important as economic resources in improving health and well-being and driving development. Research to help countries understand and overcome barriers is required in areas such as: policy, implementation and operational research; clinical research and systematic evidence reviews; disaster risk reduction and preparedness; social, behavioural, anthropological and community research; and political and social sciences.

Multistakeholder and cross-sector partnerships are critical drivers of change. In the United Republic of Tanzania, the White Ribbon Alliance for Safe Motherhood united civil society members, health professionals, academics, donors and United Nations (UN) partners in a successful three-year campaign to improve access to comprehensive emergency obstetric and newborn care at health centres.

Effective global partnerships can catalyse and support country efforts. For example, the Every Woman Every Child movement attracted more than US$60 billion dollars to women’s and children’s health between 2010 and 2015, with commitments from over 300 partners.^6^

The movement has spurred partnership mechanisms to support country-led implementation of the global strategy (2016–2030) – including the Global Financing Facility in support of Every Woman Every Child, the Innovation Marketplace, Unified Accountability Framework and the UN system’s health agencies’ H6 partnership.^1^

The global strategy (2016–2030) recognizes that human rights and other fundamental development principles – such as equity, community ownership and development effectiveness – are drivers of transformative change.^1^

In Peru, principles of equity underpinned a programme of poverty mapping to identify and prioritize reaching poor, rural and indigenous populations with social protection programmes and culturally appropriate, affordable care.^7^ In Kenya, the institutionalization of human rights principles is benefiting women’s health following complaints alleging systematic violation of women’s reproductive health rights in health facilities.

The global strategy (2016–2030) provides knowledge for integrated actions both within the health sector and with other sectors, based on country experience and current evidence. With its accompanying operational framework, the strategy serves as a roadmap for collective action to advance the health and well-being of women, children and adolescents, which will be central to achieving the SDGs.
